# Supplementary material for: Development of the early-life gut microbiome and associations with eczema in a prospective Chinese cohort
Source: mSystems. 2023 Aug 30;8(5):e00521-23. doi: 10.1128/msystems.00521-23 (PMC10654104; doi:10.1128/msystems.00521-23)
Supplement: Supplemental figures — Figures S1 to S4. [file msystems.00521-23-s0001.pdf]

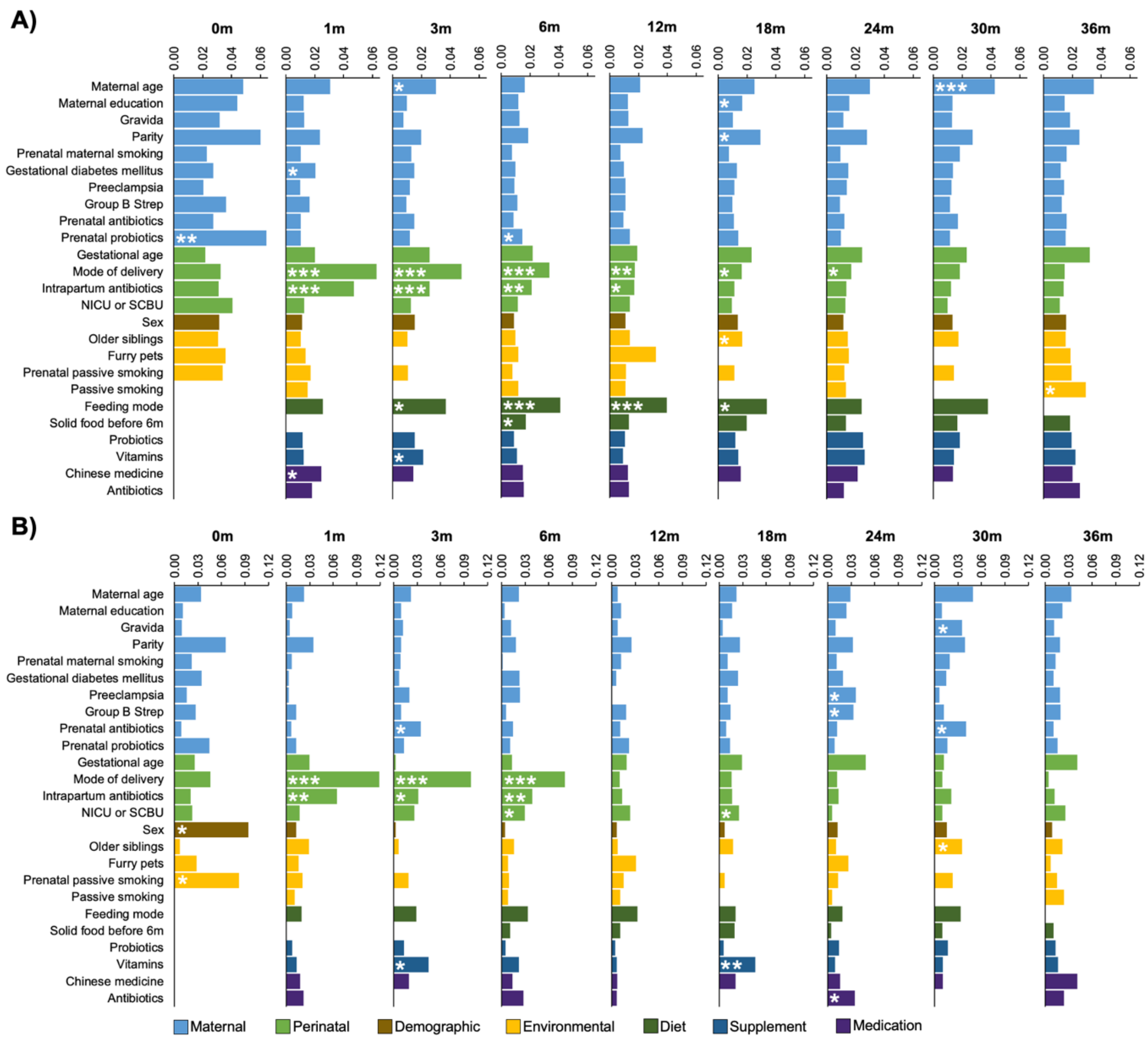

Figure S1. Effect size (R^2) of 25 variables on the gut microbiota composition across the first three years of life as determined by PERMANOVA based on unweighted (A) and weighted (B) UniFrac distances without controlling for multiple comparisons. Bars were coloured according to the categories of the variables. Effect size was not calculated when data were not collected or when the variable displayed limited variance. Groups within each variable are detailed in Table S2. NICU, neonatal intensive care unit; SCBU, special care baby unit. *P < 0.05, **P < 0.01, ***P < 0.001.

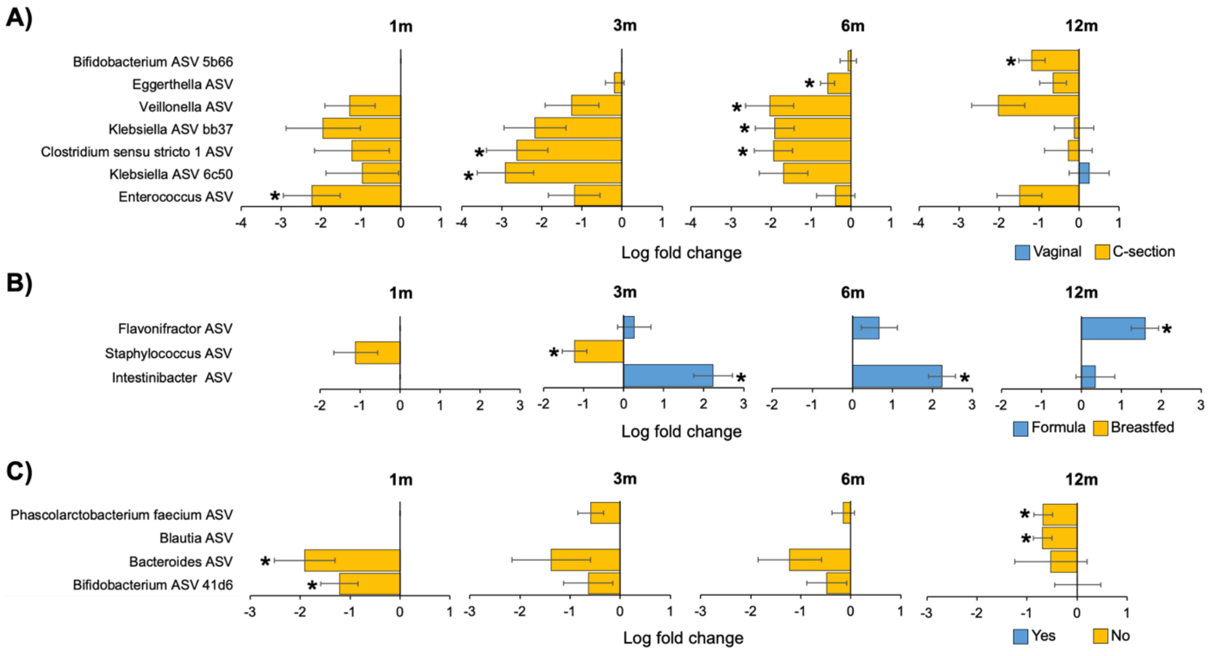

Figure S2. Differentially abundant bacterial amplicon sequence variants (ASVs) of the gut microbiota in the first year of life according to mode of delivery (A), feeding mode (B) and intrapartum antibiotics use (C). For calculations on a certain variable, the other two variables were included as covariates in the model. Length of bars represents the effect size (log fold change). Bars were coloured according to the group in which a particular ASV was enriched. Only ASVs showing significant difference at at least one time point were included here. Error bars are standard errors. *, False discovery rate (FDR) < 0.1.

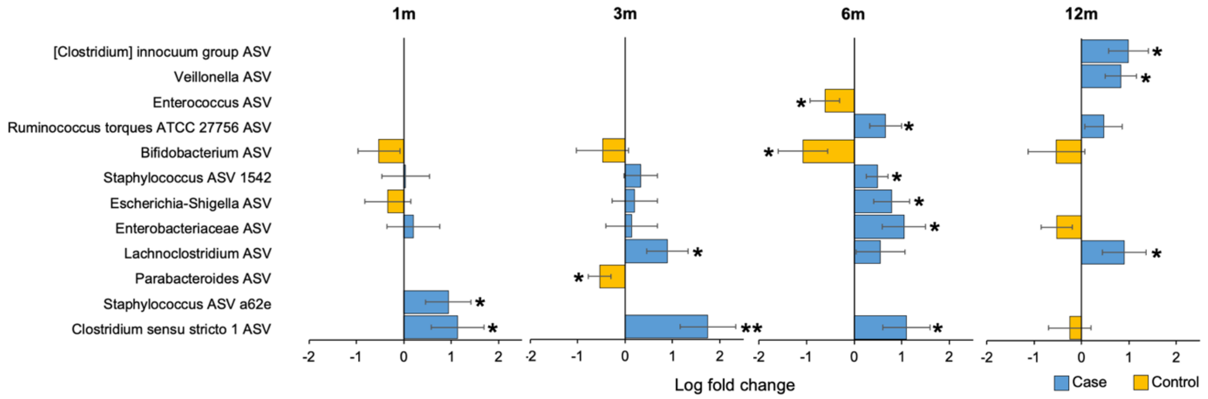

Figure S3. Differentially abundant bacterial amplicon sequence variants (ASVs) of the gut microbiota in the first year of life between subjects with physician-diagnosed eczema at 12 months after birth (cases) and those who never developed eczema by 12 months (controls). Length of bars represents the effect size (log fold change). Bars were coloured according to the group in which a particular taxon was enriched. Only taxa showing significant difference at at least one time point were included here. Error bars are standard errors. *P < 0.05, **P < 0.01.

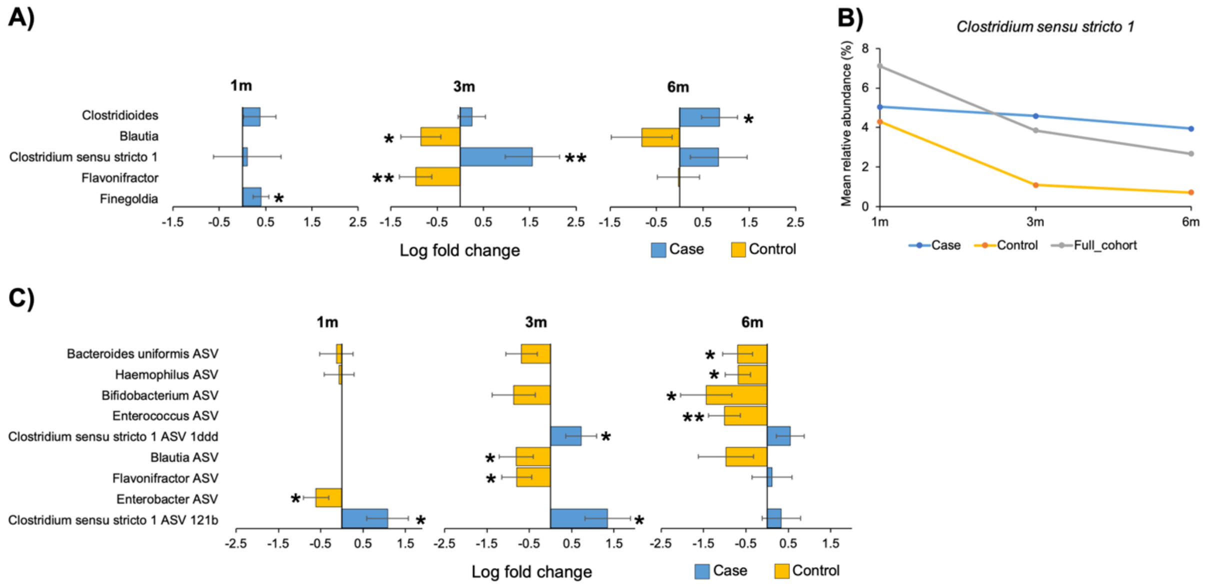

Figure S4. Nested case–control study of the gut microbiome in eczema across the first six months of life. Differentially abundant bacterial genera (A) and amplicon sequence variants (ASVs) (C) of the gut microbiota in the first six months of life between subjects with physician-diagnosed eczema at six months of age (cases) and those who never developed eczema by the same age (controls). Length of bars represents the effect size (log fold change). Bars were coloured according to the group in which a particular taxon was enriched. Only taxa showing significant difference at at least one time point were included here. Error bars are standard errors. *P < 0.05, **P < 0.01. **(B) Trajectory of the mean relative abundance of the genus *Clostridium sensu stricto 1* in the cases and controls during the first six months of life.** Data from the full cohort are also included as reference.
